# A Guided Online and Mobile Self-Help Program for Individuals With Eating Disorders: An Iterative Engagement and Usability Study

**DOI:** 10.2196/jmir.4972

**Published:** 2016-01-11

**Authors:** Martina Nitsch, Christina N Dimopoulos, Edith Flaschberger, Kristina Saffran, Jenna F Kruger, Lindsay Garlock, Denise E Wilfley, Craig B Taylor, Megan Jones

**Affiliations:** ^1^Ferdinand Porsche Distance Learning University of Applied SciencesViennaAustria; ^2^Ludwig Boltzmann Institut Health Promotion ResearchViennaAustria; ^3^Department of Psychiatry and Behavioral SciencesSchool of MedicineStanford UniversityStanford, CAUnited States; ^4^Health Promotion Fund Austria, Austrian Public Health InstituteViennaAustria; ^5^LanternSan Francisco, CAUnited States; ^6^Department of PsychiatrySchool of MedicineWashington UniversitySt. Louis, MOUnited States; ^7^mHealth InstitutePalo Alto UniversityPalo Alto, CAUnited States

**Keywords:** usability study, engagement, adherence, dropout, digital health intervention, online program, self-help, eating disorder, mobile application

## Abstract

**Background:**

Numerous digital health interventions have been developed for mental health promotion and intervention, including eating disorders. Efficacy of many interventions has been evaluated, yet knowledge about reasons for dropout and poor adherence is scarce. Most digital health intervention studies lack appropriate research design and methods to investigate individual engagement issues. User engagement and program usability are inextricably linked, making usability studies vital in understanding and improving engagement.

**Objective:**

The aim of this study was to explore engagement and corresponding usability issues of the Healthy Body Image Program—a guided online intervention for individuals with body image concerns or eating disorders. The secondary aim was to demonstrate the value of usability research in order to investigate engagement.

**Methods:**

We conducted an iterative usability study based on a mixed-methods approach, combining cognitive and semistructured interviews as well as questionnaires, prior to program launch. Two separate rounds of usability studies were completed, testing a total of 9 potential users. Thematic analysis and descriptive statistics were used to analyze the think-aloud tasks, interviews, and questionnaires.

**Results:**

Participants were satisfied with the overall usability of the program. The average usability score was 77.5/100 for the first test round and improved to 83.1/100 after applying modifications for the second iteration. The analysis of the qualitative data revealed five central themes: layout, navigation, content, support, and engagement conditions. The first three themes highlight usability aspects of the program, while the latter two highlight engagement issues. An easy-to-use format, clear wording, the nature of guidance, and opportunity for interactivity were important issues related to usability. The coach support, time investment, and severity of users’ symptoms, the program’s features and effectiveness, trust, anonymity, and affordability were relevant to engagement.

**Conclusions:**

This study identified salient usability and engagement features associated with participant motivation to use the Healthy Body Image Program and ultimately helped improve the program prior to its implementation. This research demonstrates that improvements in usability and engagement can be achieved by testing and adjusting intervention design and content prior to program launch. The results are consistent with related research and reinforce the need for further research to identify usage patterns and effective means for reducing dropout. Digital health research should include usability studies prior to efficacy trials to help create more user-friendly programs that have a higher likelihood of “real-world” adoption.

## Introduction

Digital health technologies are increasingly common and are used in the prevention, diagnosis, and treatment of mental health problems. In the eating disorders field, several online programs have been developed and demonstrate promising results [1-3]. Available evidence for these programs suggest significant improvements in preventing and treating eating disorders [2,4,5], yet poor adherence and high dropout rates remain common and challenging problems in most studies [4,6,7]. Inconsistent measures of program usage and dropout across studies contribute to high variability in interpretation of adherence and findings [2,7-10].

To date, there is scant research examining the specific reasons for dropout and poor user engagement in online programs. The multidimensional nature of user engagement complicates research design because engagement includes individual users’ thoughts and feelings, degree of activity, and attitudes towards technical aspects of the program including aspects of usability and appeal [11]. User engagement is also inextricably linked to the usability of a program [11], which refers to aspects of effectiveness, efficiency, and satisfaction [12]. Fortunately, established methods to examine program usability exist, which can also be harnessed to evaluate user engagement issues of digital health interventions.

This study aimed to explore and reveal different usability and engagement issues in the course of the redesign of the Healthy Body Image Program (HBI) [13-17], which is an evidence-based, guided online intervention for individuals with body image concerns or disordered eating symptoms, prior to its implementation. We conducted a usability study including qualitative interview elements focusing specifically on engagement. We applied a mixed-methods approach to investigate the first phase of interaction because research on this early stage of engagement is rare [18] and critical to outcome. This interaction time-point is important for determining future usage patterns and possible dropout reasons. The results of an iterative usability study exploring potential users’ initial phase of interaction with a prototype of a guided online self-help program are presented. This study also seeks to describe the method and demonstrate the value of investigating engagement issues within a usability study prior to program launch.

## Methods

### Participants and Recruitment

Participants were recruited through Web-based and print advertisements (eg, flyers, email listserve announcements) in the San Francisco Bay Area and on the campus of a large private university. Participants were offered free access to the online program and gift cards of US $10-30 depending on how much time they spent in the usability testing. The HBI program was originally designed for college-aged females, so this study also included women aged 18-25 years with an interest in improving body image and reducing disordered eating behaviors. Interested individuals were first contacted via phone in order to explain the study procedure and to conduct a short telephone screening, for which we used the SCOFF questionnaire [19]. SCOFF (Sick, Control, One stone, Fat, Food; the acronym comprises the questionnaire’s 5 items) is a widely used and well-validated eating disorder symptom screen. Consistent with prior research using the SCOFF as a screening tool, the indication of a possible eating disorder diagnosis, as measured by a positive response to 2 or more of 5 questions, was an additional inclusion criterion for this study. Thus, we ensured that the participants were representative of the individuals who are usually directed to use this particular HBI track (described below). As this study was part of a larger intervention study, the following exclusion criteria applied: lack of English language fluency, hearing impairments, and participation in any depression or anxiety intervention research study.

The complete sample of this study consisted of 9 participants. Based on an iterative usability study design approach [6,20], we aimed to conduct tests in two rounds with no more than 5 participants per round, since usability testing with 5 users reveals 85% of usability problems and more than 5 users would produce repetitive information [21]. In the first round, 4 participants tested the prototype on the computer, after which major issues were addressed. In the second round, another 5 participants tested the revised and improved program, based on the results of the first round, as a mobile app on a smartphone. The transition from the prototype on the computer to mobile app was planned as a further step in the development cycle of the program and was thus directly factored into the research design. The intervention was ultimately intended to be used on mobile and Web. Of note, the intervention was designed mobile first, even though the intervention was first accessible on the Web.

Each participant used a prototype of the program in a usability testing session, which lasted from 45-110 minutes. The time it takes users to engage with the program and to answer questions naturally varies due to the nature of usability evaluation. Thus, we followed the timing and pace of each participant to better understand individual differences. Each test had participants use the program while performing the think-aloud technique, followed by a semistructured interview and a short questionnaire. The testing sessions were conducted from August through October 2014 and took place at Stanford University School of Medicine, except three tests, which were held in other public places (eg, separate room in a library, backyard of a cafe) chosen by the participants. The privacy of the participants and a possible impact regarding their answers was taken into consideration by making sure that there were no people in close vicinity. We obtained human subjects approval from the Institutional Review Board at Stanford University.

### The Intervention

HBI begins with a Web-based assessment to determine the severity of disordered eating symptoms and risk for developing an eating disorder. Based on assessment results, participants are directed to one of several tracks of a tailored online evidence-based intervention, referred to by variations of “Student Bodies,” which is for individuals across the eating disorder risk and diagnostic spectrum [22]. Tailoring is done at the level of program assignment. This involves using the initial assessment to determine whether the intervention is suitable for the individual participant. The personalization occurs in several additional ways. First, participants select preferences, set goals, and receive dynamic feedback and recommendations based on interaction sequences. Second, each participant interacts with a personal coach and receives unique messages and feedback to support engagement and personal relevance.

In the current study, which is related to the redesign of the program, we used an offline-prototype of the guided self-help program Student Bodies–Eating Disorders (SB-ED), which is specifically designed for individuals who screen positive for a clinical or subclinical eating disorder as defined in the *Diagnostic and Statistical Manual of Mental Disorders*, 5th Edition (DSM-5), excluding full-syndrome anorexia nervosa. As part of a dissemination partnership and technology transfer, the Healthy Body Image Program and variations of “Student Bodies” were licensed to a private company, Lantern, which now provides the programs under its name. SB-ED aims to reduce disordered eating behaviors (eg, restrictive eating, binge eating, compensatory behaviors), improve body image, and support the development of effective coping skills. It includes daily sessions based on motivational principles of Cognitive Behavioral Therapy (CBT), Motivational Interviewing, and the Fogg Behavior Model for Persuasive Design (FBM) [23]. Additionally, we used the Supportive Accountability Model of guidance in Internet interventions [24] for coach-related motivational design.

**Figure 1:**
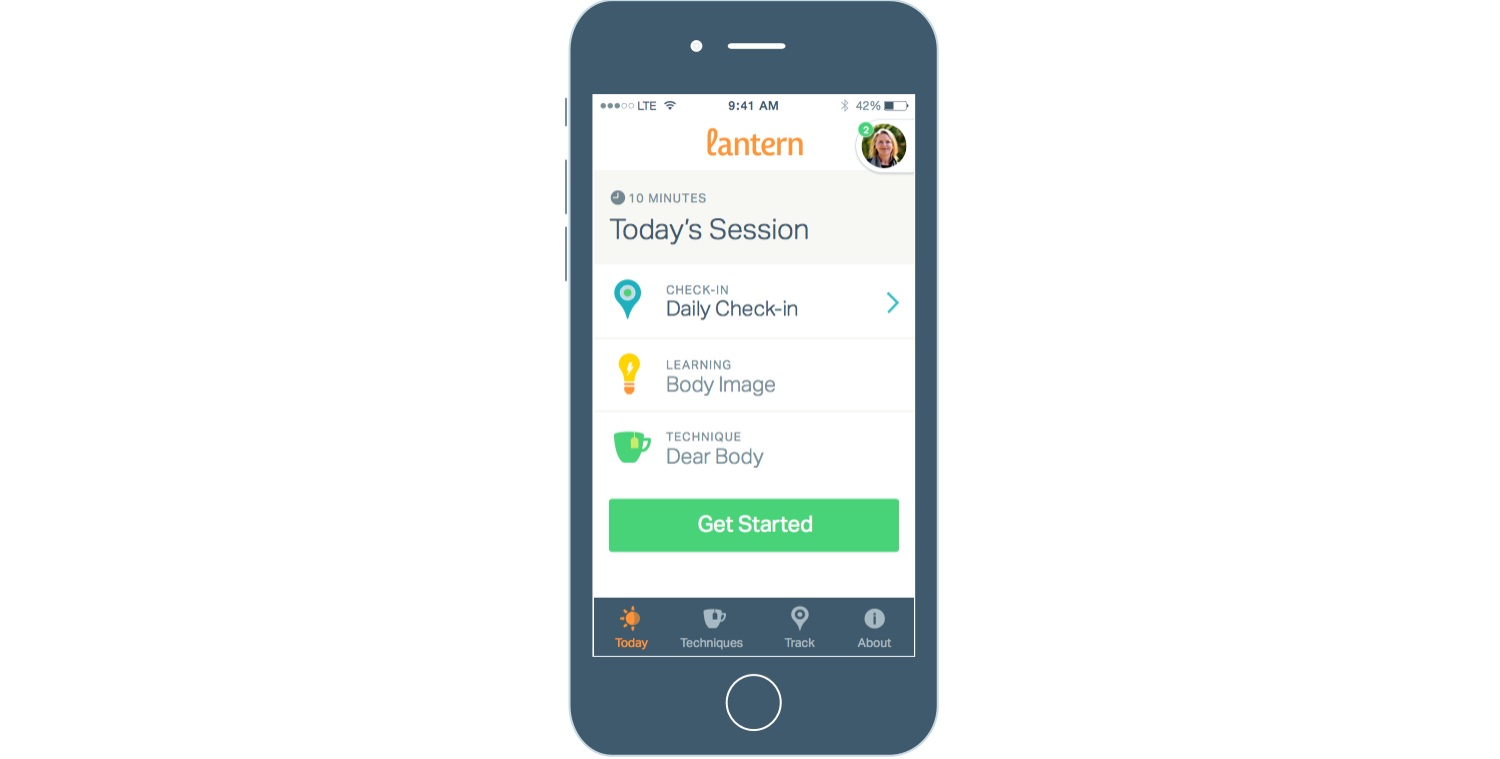
Mobile app of the Student Bodies–Eating Disorders program.

**Figure 2:**
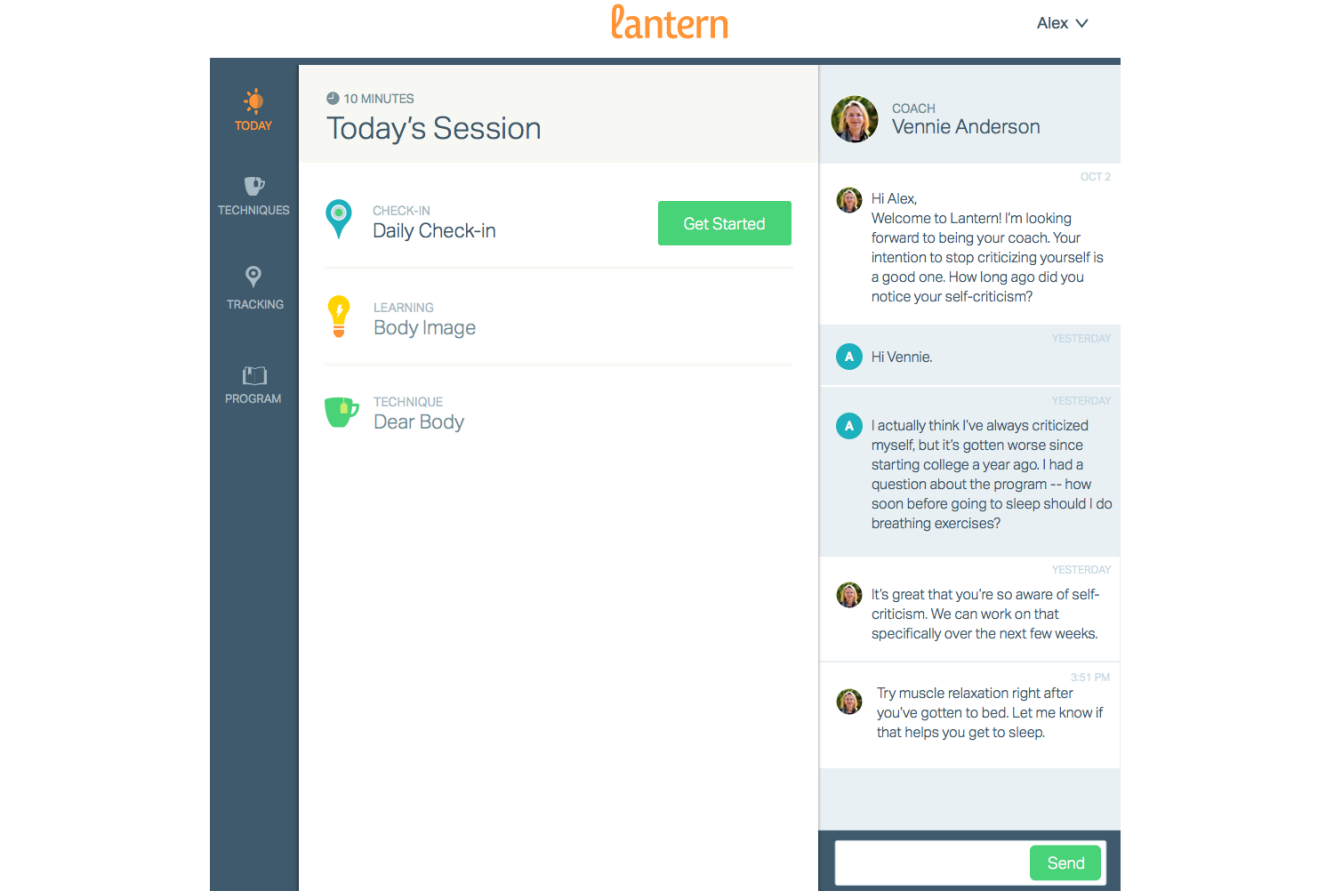
Integrated messaging function of the Student Bodies–Eating Disorders program.

SB-ED includes personal one-on-one in-app and phone-based coaching and is accessible via a mobile app (see Figure 1) and Web-based program. The user can connect with a personal coach through an introductory phone call and unlimited in-app messaging (see Figure 2). The platform includes clinical management, risk management, and quality assurance tools to support effective coaching. SB-ED includes 40 sessions lasting approximately 10 minutes each which, in the context of research, are accessible for 8 weeks. The sessions consist of a daily check-in to track eating habits and compensatory behaviors over the last 24 hours, other self-monitoring tools, psychoeducational learnings, interactive multimedia tools (eg, audioguided exercises, interactive tools), and CBT techniques.

### Data Collection and Analysis

#### Think-Aloud Task and Semistructured Interview

In each individual usability testing session, following informed consent, participants were first asked to think aloud while using the program. The think-aloud procedure involved two tasks: (1) completion of the online assessment and (2) completion of the first session of the SB-ED program. The procedure was pretested on an individual, who is not included in the 9 potential users. Participants were given an opportunity to practice the think-aloud technique by completing an Internet-based task, which was unrelated to the SB-ED program, before the usability testing started. The moderator guided the participant through the testing session by presenting the tasks and interrupted the process only if the interviewee appeared to be having difficulties thinking aloud, such as by prompting, “Tell me what you’re thinking,” “What are you looking at?,” or “What’s on your mind?” An observer recorded all comments and problems that the participants encountered. Additionally, video-analytic software (ScreenFlow) was used to capture the computer and mobile phone screen display as well as the verbal and non-verbal reactions of participants.

Next, a semistructured interview was conducted and audiotaped in order to explore important issues regarding usability and engagement. The research team developed a semistructured interview guide that included questions about the participants’ experience with the transition from the assessment to the program, general navigation issues, interaction with the coach, motivation for completing the program, and whether the user would recommend the program to others.

The think-aloud tasks and interviews were transcribed verbatim including the non-verbal reactions of the participants according to the video recordings and the notes of the observers. We coded and organized the transcripts using Atlas.ti and Excel software. Since our main aim was to identify all emerging issues and the relations between the themes, we applied thematic analysis [25]. Two researchers coded the transcripts independently by identifying themes and their relevant characteristics (categories). The themes and categories were discussed and a coding framework was created, reviewed, and interpreted by the research team. In this research context, thematic analysis seemed to be the most suitable method to combine and analyze both the think-aloud tasks and the semistructured interview.

#### Questionnaire

Finally, participants were asked to fill out a questionnaire, which included the System Usability Scale (SUS) [26], which is a standardized 10-item Likert scale questionnaire to assess a system’s usability with 5 response options ranging from “5=strongly agree” to “1=strongly disagree” (see Multimedia Appendix 1). The participant’s scores for each item need to be converted to a new number (for items 1, 3, 5, 7, and 9, the score contribution is the scale position minus 1, and for items 2, 4, 6, 8, and 10, the contribution is 5 minus the scale position), summed, and then multiplied by 2.5 to convert the scores to 0-100 [26]. A SUS score of above 68 points would be considered as above average [27]. Additionally, participants were asked questions on sociodemographic and Internet use characteristics as well as psychological treatment questions.

Following a mixed-methods approach, the SUS questionnaire data were used to validate and complement the qualitative results. Questionnaire data were analyzed in Microsoft Excel using descriptive statistics. Due to missing values, the questionnaire data of one participant had to be excluded from the analysis.

## Results

### Participants

According to our inclusion criteria, all 9 participants were women aged 18-25 years. Due the prescreening phase, all participants gave a positive response to 2 or more of 5 questions from the SCOFF questionnaire [19], thus indicating a possible eating disorder diagnosis. One participant stated she had been given an eating disorder diagnosis and currently received psychological treatment. The sample consisted of 3 high school students, 3 participants with a Bachelor’s degree, one participant with a Master’s degree, one with a college degree, and one participant did not fill out this particular section of the questionnaire.

### Questionnaire

On average, participants were satisfied with the overall usability of the program, which resulted in an average SUS score of 77.5 for the first test round and improved to 83.1 out of 100 points for the second test round. Both mean values are above the general average SUS score of 68 points [27]. The SUS is not designed to interpret individual items [26], thus, only the aggregate scores presented in Multimedia Appendix 1 were considered in the analysis.

### Think-Aloud Task and Semistructured Interview

Despite the fact that not all improvements were implemented between the test runs, the rating of the interview question about recommending the program to others improved, from 6.5 for the first round to 7.9 out of 10 points for the second round (means based on verbal rating: 0=not at all likely, 10=extremely likely).

The following analysis of the think-aloud task and the semistructured interview revealed more nuanced results. We identified five central themes consisting of several categories, which are relevant in terms of engagement and usability, across all tested program stages (assessment, transition, Session 1) and participants. Whereas the themes layout, navigation, and content point to the usability aspect of the program (see Table 1), the themes support and engagement conditions mainly focus on user engagement issues.

**Table 1:** Major usability issues and resulting changes.

Central theme	Problem description	Resulting changes
Navigation	There was confusion about how to contact the coach.	Information on how to contact the coach and the possibility of contacting the coach before program start was added to the introduction of Session 1
	Symptom self-report scales with values were not clear enough and selection buttons did not function.	Values were made more apparent and technical problems were fixed.
	Confusion about how to revisit content or check past entries.	Icons were changed to make functions more obvious.
	Difference between program and assessment tool was not clear.	Information regarding the procedure and the provider was added in the recruiting email and the introduction of the assessment tool.
	Participants felt that typing longer texts on the mobile phone is not convenient.	One exercise including typing longer texts was replaced with an interactive motivational enhancement exercise.
Content	Participants raised doubts regarding the wording of the assessment results section.	More sensitive and tentative wording was used.
	Participants did not like the exercise “Dear Thighs.”	Title was changed to "Dear Body," which had a general impact on engaging in the exercise.
	Questions were too long and/or hard to answer.	No changes possible since assessment is based on standardized instruments.
Layout	Answering format did not suit participants’ needs.	No changes possible since assessment is based on standardized instruments.

#### Layout

The majority of participants liked the layout of the program and described it as “friendly,” “youthful,” and “pretty,” similarly emphasizing the “nice colors and graphics” as well as the “easy format.” A few users mentioned that the interface seemed familiar to them and that it looked like a start-up, which was interpreted positively by some, and negatively by others, as it seemed to “be just another algorithm.” Additionally, a few skeptical comments were made, most of which concerned the questions included in the assessment, which did not suit the needs of some participants. For example, one participant suggested providing an open-ended answering format instead of a closed one.

#### Navigation

The navigation of the program was described positively for its simplicity, intuitivism, interactivity, and guidance. A few negative comments were made concerning select technical issues, for example, symptom self-report scales with values that were not clear enough or selection buttons that did not function. There was also some confusion about certain functions, such as how to revisit content, check past entries, or contact the coach in the first round of testing. The critical issue about how to contact the coach was improved before the second round by introducing the coach and the possibility of contact before the program start, after which the remaining participants had no problems finding and contacting the coach.

Comments concerning the transition from the assessment tool to the actual program were mixed. Some participants found the transition easy and clear, while others thought that the difference between the program and the assessment tool was not clear enough. This issue was improved before the second round by adding an explanation regarding the process and the provider in the recruiting email and in the introduction of the assessment tool.

In contrast to the first test round with the computer, some specific problems regarding typing longer text passages emerged during the second test round with the mobile app. Some participants mentioned that typing on the mobile phone is not as convenient and would take longer. One participant even “felt like the language was stifled on the phone,” which might influence users’ motivation for the program. As a consequence, an exercise that included typing longer texts was removed and replaced with an interactive motivational enhancement exercise.

#### Content

Participants had a general positive impression of the program and liked the repetitive encouragement provided, the “holistic” approach referring to the CBT approach, and the focus on “positive psychology” and “self-awareness.” In terms of specific content, participants had concerns about the questions on the assessment. Some participants criticized them for being too long or for being unclear regarding reference points or definitions. In spite of this, participants seemed to recognize the questions as important.

Participants’ views were especially mixed about information on the topic of “body image.” Some participants described it as “educating” and “promising” and identified with the program content related to body image. Others mentioned that it “reads like any other eating disorder website” and that they would not read it since they felt they knew everything about body image already. The journal feature to track daily eating behavior was mainly seen positively, since “there is no calorie counting” and it “teaches [users] to track in a healthy way.” Additionally, the use of a CBT approach was seen as positive and participants described it as “fancy” and “fascinating.”

Two major issues resulted from the first test round and were changed before the second test round. Some participants raised doubts regarding the text passage showing the results of the assessment tool. They felt “shocked,” “concerned,” or “scared,” and they expressed “moments of unease.” They suggested avoiding strong or serious language and instead giving the user more personal and tentative feedback. As a consequence, we used more sensitive language for the assessment results. After the wording had been changed and information had been added that the survey was not meant as a diagnostic tool, most participants in the second round agreed with their results and stated that they found it “useful” and “helpful to hear that you need help.” However, some participants noted that there was “too much” text on the assessment results. Second, unfavorable wording seemed to be the issue with the exercise “Dear Thighs,” which prompted participants to write a letter to a particular body part. Some participants were turned off by the exercise, saying that it felt “weird,” “awkward,” “crazy,” and “cheesy”, “like they were in middle school” and that they just wanted it to be over. However, when the exercise was renamed to “Dear Body,” there seemed to be a dramatic change in participant reaction and most participants found the exercise “extremely helpful,” “powerful,” and “clever.” Other minor wording issues occurred throughout the test: participants mentioned that some information was missing or that some words would benefit from an additional definition (eg, diet, meal restriction, peer).

#### Support

During the test, support in general, and more specifically, the support of the coach turned out to be major issues. All participants liked the idea of having a coach and perceived that the coach would be there to advise, help, and motivate them. This impression was made without participants’ engaging in an active conversation with the coach (as this was not possible in the prototype they tested). There was confusion about the method of user-coach communication. With this knowledge, after the first test round, the concept and role of the coach was further clarified (see the details on navigation below).

Although participants liked the idea of the coach in general, they expressed mixed feelings about having the support of an online coach. One person mentioned that “some people prefer interaction with the computer” because they might feel embarrassed when talking to a real person about their problems. Other participants mentioned that it is more “convenient,” “accessible for everyone because technology is omnipresent,” and also “important, because other resources on campus are scarce.” On the other hand, the majority of the participants mentioned that they would prefer to work with a coach in person rather than online or on the phone and that “online coaching can just be a support for personal counseling” indicating a belief that online coaching would not be sufficient on its own.

Besides the importance of the coach, some participants also highlighted the value of community support. Some participants mentioned interaction with other users who had already finished the program as being a great motivator, which may be integrated in the future.

### Engagement Conditions

For engagement, intrinsic motivation and external motivators (eg, program features) were highly relevant. In terms of intrinsic motivators, the severity of disease seemed important. Some participants raised doubts that the “program is not for people with serious eating disorder issues,” the “matter needs to be treated more seriously,” and that the program would need “to be more interactive to tackle the complex issue of eating disorders.” On the other hand, the program was seen “as a good start for people without an official diagnosis.” Other intrinsic motivators were curiosity, fun, or doing it for their family.

Time was identified as another major factor influencing likelihood of use. Generally, most participants were skeptical about the time they had to invest, stating that a daily commitment of 10 minutes was too long. They felt that it would be “too intensive for students” because they perceived that students really just “want a quick fix for their problem.” Moreover, some participants mentioned that they felt like stopping during the assessment or the exercise “Dear Thighs” since it felt too long or it made them feel uncomfortable. One person who was undergoing therapy for an eating disorder stated that 10 minutes would not be enough to tackle her problem. Conversely, some participants found the procedure and the exercises engaging and the overall time commitment was fine.

Another important factor in terms of finishing the program was the question of trust, which was challenging in an online format where there was initial confusion about the nature and role of the remote coach. However, credibility in terms of having already heard about the program or its developers had a positive impact. Additionally, information about the program developers was added in the recruiting email before the second test round.

Effectiveness or success was seen as a central motivator because participants mentioned that they would finish the program if they were to see “improvements,” “results,” or a “gain in health.” For instance, it was expected that the program or the coach would provide external motivation by integrating daily reminders, motivational pop-ups, affirmations, tips and advice, and detailed and customized feedback. Some participants also seemed to be impressed by the number of students enrolled in the program, the rate of symptom reduction (50%) in disordered eating behavior after completing the program, and the research background, which was outlined on the registration page. Anonymity and privacy were other factors that were highlighted positively and mentioned as relevant for program completion*.* Hence, no issues were raised regarding the confidentiality of the data. In addition to the program’s affordability, additional incentives were discussed, such as “a gift card together with personal commitment would seal the deal” and suggestions that program use should be compensated with class credits.

In some cases, it was also misread as a fitness or weight management tool. More information was needed about how the assessment questions relate to the program personalization and other program abilities, such as customization of questions or if the program “learns” from the users’ answers and “how the program gets to know the people.” Some participants also indicated that they had not read or paid attention to the introduction text.

Participants reported mixed feelings about using the program on their mobile phone or via the computer, yet the majority of the participants said that they would rather use it on their mobile phone as a mobile app or a widget. However, participants also mentioned some possible pitfalls to this, such as “it is tiring to read long texts on the phone,” that “texts are rather skimmed than read,” and that “less scrolling would be better.” Whereas the majority of participants described the mobile app as convenient, some participants also said that they would probably forget to use the app and others mentioned that they would prefer to use it on both the computer and mobile phone.

## Discussion

### Principal Findings

This study aimed to assess and improve usability and engagement aspects of a guided online self-help program for improving body image and reducing disordered eating symptoms. The difference between the first and the second round of testing shown in the SUS as well as in the think-aloud and interview section clearly highlights the value and reliability of performing an iterative usability study using a mixed-methods approach. This study’s findings also further support the importance of incorporating usability and feasibility studies as part of the digital health intervention design process [28-30]. Usability testing is a valuable and effective method for executing a user-centered design process, illuminating end-user needs and perceptions, and facilitating intervention adaptation prior to a broad implementation.

This study found a need for intervention improvement in five major areas: layout, navigation, content, and support and engagement conditions. Regarding content, wording and language used was an important issue as it was found to trigger negative emotions in the first iteration (eg, when reading the results of the assessment and the title of the “Dear Thighs” technique). Choosing the right design, wording, and developing language in a user-centered and participatory design process is critical and may have a significant impact on engagement [31].

In terms of assessment, a number of participants had concerns about the assessment logic and assessment items. Because the items were derived from standardized instruments, it was not possible to change question text. However, other simple changes in design resulted in immediate improvement. Assessment results were presented to highlight “strengths” and “challenges” (and not diagnoses) and were intended to help participants gain perspective about their need for help and the apparent urgency and severity of their needs. The feedback provided to participants aimed to help them make an informed and empowered decision about whether the online intervention was appropriate or if they would be better served by seeking in-person evaluation and therapy (information with referral information was provided). The participant feedback on the standardized questions reveals a difficult challenge of using evidence-based and psychometric sound instruments for online assessments. Few research assessments are constructed on the basis of being user friendly, and many are developed using highly educated populations (eg, college students). In the future, assessments should be developed with consideration of how they might be used with digital programs, and text should be written at an average reading level to improve accessibility.

Concerning navigation, a major issue was that it was not obvious to participants how to contact the coach. This finding was important due to the central role of the coach and prompted addition of information and design change to help participants understand the coach’s role and how to contact their coach.

For engagement, the identified main themes center on motivation, ability (simplicity), and triggers as outlined in the FBM [23]. The anticipation that the coach provides external motivation by setting reminders or affirmations refers to the Supportive Accountability Model [24]. The coach also played an important role in promoting engagement as indicated by participants, who mentioned that the support of the coach would be a primary motivator to finish the program. This is consistent with research confirming that the inclusion of professional therapist or “technician” support improves program adherence [5,8]. In this context, the participant’s assumption that the coach would be a virtual coach was crucial for the research team, especially since the majority of the participants mentioned that they would prefer to work with a coach in person.

In terms of motivators, the users’ symptom severity or specific diagnosis seemed important, as it refers to the core motivator “pleasure/pain” in the FBM [23], indicating that the need for seeking help rises according to the personal pain or severity level of the disease. In this study, some participants doubted the program’s effectiveness in relation to their specific needs and expressed a desire for professional face-to-face therapist support. The preference for specialist treatment is in line with other recent research findings suggesting that individuals treated for anorexia nervosa prefer health professionals with high professional communication skills and an adequate knowledge of eating disorders [32]. Interestingly, Dölemeyer et al [2] found that studies exclusively enrolling participants with binge eating behaviors showed relatively low dropout rates, assuming that the motivation of this patient group is relatively high due to high psychological impairment and other related health problems. Consistent with Dölemeyer et al [2], previous studies using previous versions of the Healthy Body Image Program have also shown low dropout among participants with subclinical eating disorder symptoms and strong effects for participants with binge eating [13-17].

Some participants also seemed to be impressed by the success of other users, for example, the number of students enrolled in previous versions of the program so far and the rate of symptom reduction (50%), which was outlined on the registration page. “Seeing results” such as a reduction of disordered eating symptoms in their own lives was seen as another core motivator, which relates to the dimensions “hope/fear” in the FBM [23], which is characterized by the anticipation of an outcome. The Motivational Interviewing and Cognitive Behavioral theories also posit that expectancies influence engagement and outcome. In CBT, this originates from Bandura’s Social Learning Theory [33], which, applied to this study, could explain why testimonials improve positive expectancies. Belief in the program effectiveness and success is important for enhancing engagement and preventing dropout [10]. In the future, information from the assessment data should be customized better to the user by noting how the program has worked with individuals with similar scores.

Time, which is described as an important element of simplicity in the FBM [23], also emerged as a potential factor in engagement, since some participants viewed 10 minutes of daily use as too much time. However, the possibility of using the mobile app was seen as more convenient, and previous research has highlighted convenience as an important criterion for the use of digital health interventions [34].

Overall, the results of this study are consistent with general characteristics of digital health interventions for behavior change and self-management suggested by Murray [35]. These interventions need a strong theoretical foundation, perceived personal relevance to the user, perceived effectiveness, tailoring, persuasive technologies, credibility, social networking, and regular “push factors” (including human support or periodic prompts) in order to increase adherence.

### Strengths and Limitations

Although the concepts of usability and engagement are inextricably linked, adequate and standardized methods to investigate issues of engagement are scarce. Thus, the strength of this study is that it demonstrates that engagement issues can be investigated within the scope of a usability study. This research design proved to be effective in identifying a range of issues for improvement and facilitating measurable program improvement prior to implementation. Ultimately, the conduction of usability studies fits with the general demand for alternative methods to evaluate behavioral intervention technologies. Traditional evaluation methodologies such as randomized controlled trials are not compatible with fast-changing customer expectations and rapid technology advancement, which demand less time-intensive methods [36].

One major limitation of this study is the possibility that participants were influenced by the study situation itself, since the task of thinking aloud and simultaneously being observed might have provoked unintended reactions or statements. However, we tried to reduce this possible bias by practicing the think-aloud method with each participant prior to beginning the actual study. In three cases, the study situation might have been influenced because the tests were held in public places chosen by the participants. We tried to reduce possible influences by ensuring that there were no people in close vicinity, so that participants felt comfortable speaking openly. Another limitation was that the participants did not get individualized results based on their answers to the assessment questions since this was not possible at this stage of the development process of the program but instead a standardized results page according to their initial SCOFF results. This might have caused discrepancies regarding their expected results and thus led to negative statements about the wording of the results page. Another limitation is that participants tested a prototype rather than the actual program, so they could not use or test many of the program functionalities.

### Conclusions

Despite the limitations, this usability study allowed us to improve and refine our guided online SB-ED program prior to its launch by making changes based on our target group’s concerns and preferences. Our main findings regarding usability and engagement issues of online health programs are fairly consistent with prior research findings of similar studies, suggesting that this was a reliable and effective research method. The true advantage of conducting small-scale usability studies is evident in their ability to reveal specific program issues from the perspective of the target population in the implementation phase and at the same time contribute to larger research-based insights. Usability studies of programs incorporating online assessments or questionnaires need to pay attention to standardized question items, which cannot easily be adapted to user needs and thus can highly interfere with usability and engagement aspects. Accordingly, future usability and engagement research for different stages of digital health program use is needed in order to identify general usage and adherence patterns, which can ultimately help improve program adherence and reduce dropout.

## Multimedia Appendix 1

Individual SUS scores.

## Abbreviations

CBT: cognitive behavioral therapy
DSM-5: Diagnostic and Statistical Manual of Mental Disorders, 5th Edition
FBM: Fogg Behavior Model for Persuasive Design
HBI: Healthy Body Image Program
SB-ED: Student Bodies–Eating Disorders
SCOFF: Sick, Control, One stone, Fat, Food
SUS: System Usability Scale
